# Lesser of Two Evils? Foraging Choices in Response to Threats of Predation and Parasitism

**DOI:** 10.1371/journal.pone.0116569

**Published:** 2015-01-30

**Authors:** Janet Koprivnikar, Laura Penalva

**Affiliations:** 1 Department of Chemistry and Biology, Ryerson University, Toronto, Ontario, Canada; 2 Department of Biology, Brandon University, Brandon, Manitoba, Canada; Estacion Experimental de Zonas Áridas (CSIC), SPAIN

## Abstract

Predators have documented post-encounter (density-mediated) effects on prey but their pre-encounter impacts, including behavioural alterations, can be substantial as well. While it is increasingly evident that this “ecology of fear” is important to understand for natural enemy-victim relationships, fear responses of hosts to the threat of infection by a parasite are relatively unknown. We examined larval amphibian (*Lithobates pipiens*) foraging choices by experimentally manipulating the presence of cues relating to predator (larval odonate) or parasite (the trematode *Ribeiroia ondatrae*) threats. Tadpoles avoided foraging where predator or parasite cues were present; however, they did not treat these as equal hazards. When both threats were simultaneously present, tadpoles strongly preferred to forage under the threat of parasitism compared to predation, likely driven by their relative lethality in our study. Our results indicate that altered spatial use is an important anti-parasite behaviour, and demonstrate that parasite avoidance can affect foraging in a manner similar to predators, warranting greater study of the pre-encounter effects of this enemy type.

## INTRODUCTION

Natural enemies such as predators have important effects at different levels in natural ecosystems. The direct (i.e. density-mediated) effects of predators through consumption are well-studied, including their regulation of prey populations [1]. However, it has become evident that predators also have significant indirect influences which occur pre-encounter/consumption through alterations of prey behaviour, physiology, and vulnerability to infectious diseases [2,3], and these may collectively approach the magnitude of consumptive effects—see review by [4]. Foraging is one type of behaviour that is often modified in prey perceiving a threat of predation. To avoid predators, prey typically decrease their level of foraging, alter their spatial use (including abandonment of high resource patches), and may shift the timing of their foraging [5,6,7]. These changes in foraging can have important consequences that outweigh those directly affecting growth, survivorship, or fecundity [8]. For example, the presence of wolves causes elk to alter various behaviours including their foraging levels and diet choices, which are then associated with decreased calf production and reduced population size [2].

Parasites are also common natural enemies that have substantial impacts on individual hosts, populations, and communities [9,10,11]. Although the means by which parasites affect hosts differs from that of free-living predators [12], we can separately consider their effects before and after infection to differentiate between pre- and post-encounter influences, respectively. These are roughly analogous to the non-consumptive and consumptive effects of predators but not all parasite life history stages (e.g., cysts) consume host energy/tissue, thus pre- and post-encounter are more generally applicable terms when comparing a broad suite of natural enemies. While there are many potential similarities between predators and parasites, most studies have focused on post-encounter effects related to parasitism following the demonstration that both natural enemies can regulate prey/host populations in a comparable manner [13]. In contrast, the pre-encounter effects of parasites are relatively unknown despite recent efforts to integrate this enemy type into a general study of the ecology of fear—see review by [12]. Because encounters with predators are generally considered more likely to have lethal consequences compared to parasites [14], the resulting assumption has been that animals should show stronger avoidance behaviours in response to a predation threat relative to parasitism [12]. However, many animals engage in various behaviours to lessen their chances of becoming parasitized, including avoidance of infected conspecifics for directly-transmitted pathogens, grouping to dilute ectoparasite attacks, and altered microhabitat use—see reviews by [15,16,17]. Strong anti-parasite behaviours should be favoured if they decrease the costs of immunological defences and direct damage from parasites [15] but could have significant pre-encounter effects analogous to those incurred by predators. Despite this possibility, there have been few studies of this nature to date [18,19,20,21].

Post-encounter (i.e., after infection), hosts often exhibit behavioural alterations, including loss of fear and altered levels and timing of activity [22]. Foraging by infected animals can also be affected. For instance, gerbils infested with fleas are more likely to abandon high-resource patches and spend less time foraging in favour of increased grooming [23]. But altered foraging is also an effective way to avoid becoming parasitized to begin with [15,16,24]. Various hosts avoid consuming infected prey or foraging near feces containing parasite infectious stages, and small mammals will forego foraging in areas with high ectoparasite density [25,26,27]. While these examples demonstrate the potential pre-encounter effects of parasite avoidance, how does fear of this natural enemy directly compare to that of predators with respect to foraging choices? Notably, behavioural responses to threats posed by different types of natural enemy should correspond to the risk that they pose [28,29].

Larval amphibians provide a good model system for examining the pre-encounter effects of predators and parasites as they have been demonstrated to exhibit defensive behaviours in response to both types of natural enemy. In the presence of predators such as larval odonates and fish, tadpoles usually alter the levels and timing of their activities, seek refuges, and abandon high resource patches [30,31,32]. With respect to pathogen avoidance, adult amphibians avoid laying eggs in water bodies containing snails that are host to trematode (flatworm) parasites, and tadpoles avoid contact with conspecifics infected by a directly-transmitted pathogenic yeast [33,34]. In addition, many larval amphibians display strong anti-parasite behaviours after contact with free-living trematode infectious stages (cercariae), including elevated activity and twisting, presumably to evade or remove these parasites before they penetrate [35,36,37,38]. However, these responses are short-term and tadpoles seem to show no overall change in activity level when exposed to cues representing ongoing trematode presence compared to those signaling a threat of predation [21]. Consequently, it has been suggested that larval amphibians may have few pre-contact options for avoiding these common macroparasites [21]. If we consider tadpole anti-predator behaviours, spatial avoidance is common and thus also likely to be an effective tactic for avoiding parasites. Larval amphibians will distance themselves from areas where infected snails or cercariae are present, notably to the same extent as areas containing predation cues [19], although these were not directly compared as choices in an earlier study.

We investigated the foraging choices of tadpoles when offered food with or without natural enemy cues present (larval odonate predator or trematode cercariae), as well as when the only option was to forage with both threats at hand. By doing so, we aimed to answer two main questions: 1) Do larval amphibians perceive the threat posed by a pathogenic parasite and alter their foraging choices, demonstrating potential pre-encounter effects? 2) How do the pre-encounter effects of predators and parasites compare to one other in this system? While we know that predators have trait-mediated effects on tadpole behaviour and morphology [31,39], trematode parasites are an ubiquitous threat to larval amphibians [40,41] and could have important pre-encounter effects in addition to their documented post-encounter impacts. As our study is one of the only few to date examining how the threat of parasitism affects host habitat use, and is the first direct manipulative comparison of how predators and parasites affect foraging choices, these results will aid efforts to integrate studies regarding the ecology of fear.

## MATERIALS AND METHODS

### Natural enemy cues

We chose the trematode parasite *Ribeiroia ondatrae* because it infects tadpoles throughout North America and can cause high levels of mortality and pathology by inducing limb malformations, although these outcomes are dependent on host species and developmental stage, as well as parasite dose—see review by [40]. This trematode uses planorbid snails as 1^st^ intermediate hosts in which free-swimming infectious stages (cercariae) are produced and emerge seeking appropriate 2^nd^ intermediate hosts including fish and larval amphibians. After encysting in the tadpole, particularly around developing hind limb buds, the life cycle of the parasite is completed after this 2^nd^ host is eaten by avian or mammalian definitive hosts in which adult worms will develop [42]. We obtained *R. ondatrae* cercariae that emerged from field-collected snails kept in Petri dishes of dechlorinated water and froze them within 1.5 mL microcentrifuge tubes. Before their use on each behavioural recording day, these tubes were thawed and 25 cercariae were pipetted into separate 1.5 mL tubes with enough dechlorinated water added to attain a total volume of 1 mL.

To obtain predation-related cues, we kept larval odonates (*Anax* genus) overnight in separate 200 mL containers of dechlorinated water, removing the predators the next morning. In the morning, we euthanized two *Lithobates pipiens* (northern leopard frog) tadpoles in a 0.2% buffered solution of MS-222 and then placed them into a Petri dish with 15 mL of dechlorinated water before evisceration to simulate a predation event. As predation of conspecifics releases chemical alarm cues that larval amphibians respond to, these are commonly used in predation studies [19]. To simulate a strong predation threat, we thus combined cues from both the odonate predator and tadpoles that were “predated” upon by adding the liquid from the prey Petri dish to the predator water container. Separate 1.5 mL microcentrifuge tubes were then filled with 1 mL of this predation cue. Fresh cues for both natural enemies were generated for each of the 5 behavioural recording days (see below). While parasite threat was represented by both visual and possible chemical cues, but only the latter for predators, we chose these based on the most likely natural scenarios. In particular, larval amphibians primarily rely on olfaction to detect predation threat [43], and because the vast majority of trematode cercariae never reach their 2^nd^ host, dead infectious stages are most abundant. Tubes filled with 1 mL of dechlorinated water were used as sham additions in the no threat cue zone of the foraging choice arena.

### Foraging choice experiments

We conducted our foraging choice trials in two separate 3-armed mazes constructed of plexiglass. Each arm of the mazes was 23.5 cm long and 10.5 cm wide with a maximum depth of 9.5 cm and also had a red line drawn approximately half-way across the middle (i.e. approximately 10 cm from the far side of the arm). Consequently, each maze was divided into 4 zones: 1 and 2 referred to the arms in which enemy cues or food were placed, 3 corresponded to the centre of the maze where the 3 arms joined, and zone 4 was the most distant of the arms and did not receive any cues or food.

**Figure 1:**
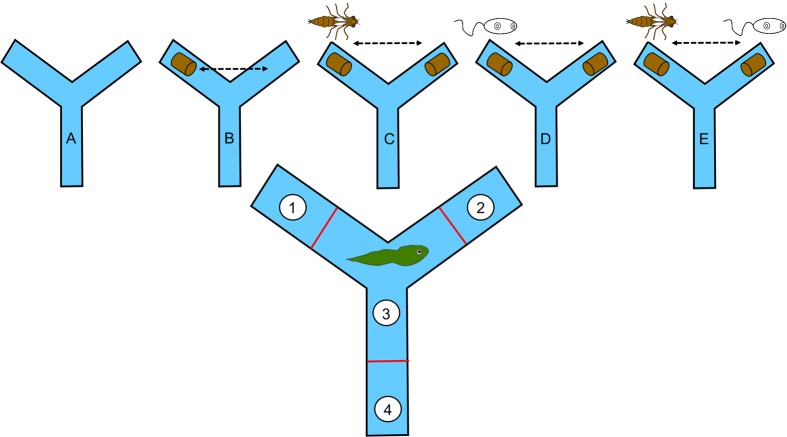
Experimental design to investigate effects of natural enemies on larval amphibian foraging choices using 3-armed mazes divided into 4 zones. Focal arms (1 and 2) had different stimuli across 5 experimental conditions: A = no food or enemy, B = food/no food, C = food with predator/no predator, D = food with parasite/no parasite, and E = food with predator/parasite.

We observed the behaviour of 20 *L. pipiens* tadpoles in Gosner developmental stage 28 [44] in 5 different experimental conditions: A = no enemy cues or food, B = food (0.5 cm long rabbit food pellet) in either zone 1 or 2, C = food in both zones 1 and 2 and predation cue in one of these zones as well, D = same as C but with parasite cue, and E = same as C and D but predation cue was in either zone 1 or 2 and parasite cue was in the other (see Fig. 1). Each tadpole was recorded on 5 consecutive days, corresponding to the 5 experimental conditions, with random assignment of treatment to day and which zone received food/enemy cues within and among individuals. However, we ensured that half of the tadpoles would receive food in zone 1 for condition B while the others had this placed in zone 2. Similarly, 50% of individuals received the predation or parasitism cue in zone 1 for conditions C and D, with equal splitting of enemy cue assignment between these 2 zones in condition E as well.

The 20 tadpoles used in this study were haphazardly chosen from aquaria containing multiple clutches that had been locally collected as eggs and raised in the lab. Those selected for this study were maintained in separate opaque 1.5 L plastic containers filled with dechlorinated water on a 14:10 light-dark cycle for 1 week prior to commencing behavioural observations. Prior to each trial, we filled the maze with 1250 mL of dechlorinated water and then added food and/or the 1 mL contents of the tubes as per the assigned experimental condition for that day 10 minutes before recording began. For conditions B-E, the arm not assigned food or enemy cue received the sham addition. Tadpoles were added to the centre of the maze (i.e., in zone 3) and allowed to acclimate for 15 minutes behind cardboard blinds that separated the mazes from each other and the experimenter. After this 15 minute period, digital recording cameras mounted on downward-facing tripods were turned on for 15 minutes. Once the recording was complete, the blind separating the experimenter was removed, the maze contents were discarded, and each tadpole was returned to its home container. Tadpoles were fed boiled organic spinach (a 1 cm^2^ piece for each) after each recording session but we removed uneaten food after 4 hours to provide motivation for foraging the next day. For each new trial, the mazes were washed with hot soapy water, dried with paper towel, and filled with fresh dechlorinated water. After 5 days when every one of the 20 tadpoles had been recorded in each of the 5 experimental conditions, they were euthanized in a buffered solution of MS-222. This study was carried out in strict accordance with the policies and guidelines of the Canadian Council for Animal Care. The protocol was approved by the Brandon University animal care committee (protocol number 2009R03–3) and all efforts were made to minimize suffering. Amphibian eggs were collected under a permit issued by the Manitoba Conservation and Water Stewardship provincial agency in Turtle Mountain Provincial Park (49° 3′16.12″N, 100° 3′43.07″W).

### Data analysis

While watching the recordings, we noted the position of the tadpole within the maze (zones 1–4 as described above) every 30 seconds. After we calculated the proportion of time points that each individual spent within the 4 zones for the 5 conditions, we performed an arcsine-squareroot transformation of the data (all proportion data from this point refer to transformed variables). We conducted a t-test to determine whether tadpoles had an inherent bias for spending time in either zone 1 or 2 in condition A (i.e., no cues or food present). To establish that tadpoles were in fact attracted to the offered food and motivated to forage, we used a linear mixed model (LMM) with proportion of time in zones 1 and 2 during condition B as the dependent variable (normal distribution and identity link function), food presence as a fixed effect, and zone in which food was placed (1 or 2) as a random effect. After finding that tadpoles had no inherent preference for either zone 1 or 2 (see Results), we did not include this factor in subsequent analyses. To examine the effect of natural enemy presence on tadpole foraging choices, we also used linear mixed models for the treatments in which threats were present (conditions C-E). We used the proportion of time points spent in zone 1 or 2 as our dependent variable (normal distribution and identity link function), with high threat presence and experimental condition as categorical fixed effects, as well as individual tadpole as a random categorical factor. High threat presence was designated as the zone containing the predator in conditions C and E (predator/no predator and predator/parasite, respectively), and the zone containing parasite cue in condition D. Treatment day was included as an ordinal fixed effect (i.e., on which of the 5 recording days each of the 3 threat conditions occurred); however, we dropped additive models containing nonsignificant main effects and interactive models containing nonsignificant interaction terms. We also examined the last foraging choice of tadpoles during the recording period (i.e., position at the 15 minute mark) for the 3 threat treatments, using contingency tables to determine whether their frequency of occurrence in zone 1 or 2 was affected by high threat presence, as well as threat treatment. All analyses were performed using SPSS 21.0. Data are available in (S1 Table).

## RESULTS

**Figure 2:**
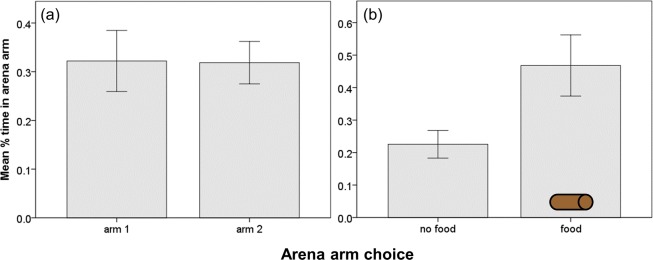
Mean proportion of time points (± S.E.) spent by larval amphibians within 3-armed choice arenas with the 2 focal arms receiving (a) no food or enemy cue, or (b) food within either arm 1 or 2.

**Figure 3:**
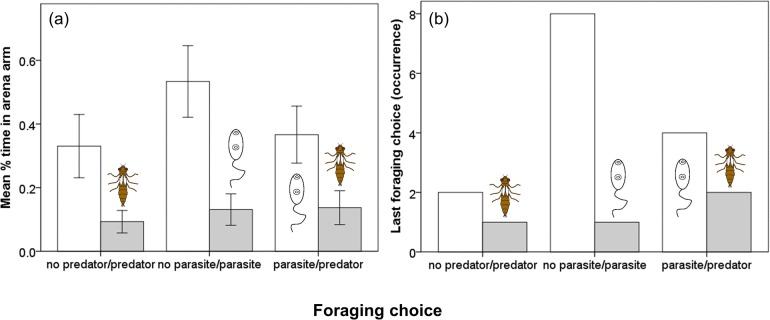
Foraging choices of larval amphibians within 3-armed choice arenas: (a) mean proportion of time points (± S.E.) in focal arms when one or both contained a natural enemy cue, and (b) last foraging choice during 15 minute recording period while in the presence of natural enemies.

The results of the t-test indicated that tadpoles had no inherent preference for either zones 1 or 2 of the mazes in the absence of food or enemy cues (*t*_38_ = 0.048, *P* = 0.962; Fig. 2a). In contrast, tadpoles spent significantly more time in the zone in which food was present, indicating that they engaged in foraging (LMM: *F*_1,36_ = 5.269, *P* = 0.028; Fig. 2b). Only natural enemy presence was retained as a significant predictor in our models (LMM: *F*_1,118_ = 21.881, *P* < 0.001) as tadpoles spent significantly less time foraging in the zone containing a high threat; however, this was not affected by experimental condition (additive LMM: high threat, *P* < 0.001; experimental condition, *P* = 0.272). In other words, tadpoles avoided foraging in the zone that we classified as containing the higher threat no matter the experimental condition (predator or parasite, respectively, when singly presented), with predator cue consistent as the greater threat even when parasite cues were simultaneously present (Fig. 3a). For the 18 tadpoles whose last position was in a food-containing zone, the frequency of their occurrence was significantly lower in the arm containing the higher threat (*Χ* ^2^ = 11.11, df = 1, *P* = 0.001) but this was not affected by the threat condition (*Χ* ^2^ = 1.29, df = 2, *P* = 0.526; Fig. 3b)

## DISCUSSION

Our results indicate that both predators and parasites can have pre-encounter effects on larval amphibians by influencing their foraging behaviour. Tadpoles avoided foraging in areas where predation cues were present but showed an equally strong avoidance response to cues representing a threat of parasitism. That tadpoles eschewed the arms of the maze containing predation threat signals is consistent with previous studies documenting spatial avoidance anti-predator behaviour in response to larval odonate- and conspecific-derived chemical cues [45,46]. The preference by tadpoles for foraging where trematode infectious stages (cercariae) were absent indicates that they are capable of perceiving the presence of this pathogenic parasite without physical contact and also consider infection by *R. ondatrae* as a threat. This is in agreement with a previous study reporting that larval toads maintained a distance between themselves and live cercariae of another species (*Echinostoma trivolvis*) which was as great as that for predation chemical cues [19], although these enemies were separately examined. Given that *R. ondatrae* is capable of causing high mortality and skeletal deformities in larval amphibians [40,42], we might expect these hosts to engage in various anti-parasite behaviours as a first line of defence. Our results add to a growing body of work illustrating altered foraging in animals in order to avoid parasites [20,26,27], indicating that both types of natural enemy have important consequences for the ecology of fear.

As well as examining how fear of predators or parasites independently affected animal foraging, we provide the first direct comparison of this behaviour through simultaneous manipulation of these two different classes of natural enemy. While the magnitude of avoidance response was similar when tadpoles were given the choice to forage with or without either enemy present, they preferred to forage under the threat of parasitism compared to predation when both feeding zones contained enemy cues. Even though parasite cues were perceived as a threat, this was apparently less than that posed by predation in our study. This is probably explained by the fact that a single encounter with a larval odonate predator is more likely to be lethal for a tadpole relative to that with 25 *R. ondatrae* cercariae as used here. While this parasite can cause larval mortality and limb malformations that hinder the feeding and escape ability of metamorphic amphibians [47,48], its pathology is dependent on dose and host developmental stage [49]. Consequently, repeated encounters with cercariae would be necessary to cause death for most tadpoles, or successful one-time infection by many cercariae during a vulnerable host developmental stage. However, host/prey responses should reflect the quantitative risks of being infected or eaten, respectively. Because the probability of encountering predators and *R. ondatrae* cercariae will vary across habitats [40,50], tadpoles may instead risk foraging in microhabitats with a low chance of predation and avoid those where many cercariae are simultaneously present. Notably, the prevalence of infection with *R. ondatrae* has been reported to be as high as 100% in larval amphibians from some field sites [40]. As we did not systemically vary the relative strength of the parasite and predator cues to determine whether there was a critical threshold causing a foraging avoidance shift, this warrants examination in future studies, as well as the importance of host life history stage.

As detailed earlier, previous studies of anti-parasite behaviours in amphibians indicate a preference for adults to oviposit where the risk of infection to their offspring is low, and tadpoles avoid yeast-infected conspecifics [33,34]. However, activity levels of larval amphibians have been the focus of most investigations relative to spatial or temporal behavioural alterations. Many tadpoles demonstrate short-term activity elevations in the presence of live trematode cercariae which are effective at reducing infection such that individuals showing no or weak responses typically have higher parasite loads [36,37,38]. In spite of the demonstrated benefits of such increased activity, larval amphibians do not seem to do so in the continuous presence of parasite cues over longer time periods even though predation cues generated behavioural and morphological responses [21]. This suggests that tadpoles do not sustain elevated activity in order to avoid trematode infectious stages before they make physical contact [21] but our demonstration of parasite avoidance through spatial means adds another possibility to their defensive repertoire. It is important to note that while altered activity and habitat use are effective parasite avoidance strategies, both can also negatively impact vital activities, increasing the likelihood of consumptive and non-consumptive effects. As well as presenting a trade-off with foraging, many anti-parasite behaviours such as conspicuous/increased activity may also conflict with others, including predator avoidance [49,51,52]. Spatial avoidance of parasites may play a particularly important role in these circumstances if activity alterations exact a higher cost and cannot be sustained.

We did not anticipate that larval amphibians would respond so strongly to dead cercariae but this has been previously reported in studies with fish [53]. Most investigations of anti-parasite behaviour in tadpoles have employed live cercariae that were able to contact tadpoles but some have prevented this with impassable mesh barriers [19,21]. Given that tadpoles still responded to live cercariae at times without direct contact, it has been suggested that chemical or vibrational cues were involved [19]. Because *R. ondatrae* cercariae have proteolytic enzymes that aid in the host penetration process [42], this parasite may evoke a particularly strong avoidance response and the presence of these chemicals was likely enhanced due to parasite freezing and thawing in our study as this would rupture the contents of the penetration glands. As dead cercariae were physically present in our study, it is not clear by which sensory means tadpoles primarily perceived this natural enemy (chemical or visual). It will be important to further study the means by which aquatic animals can detect a threat of parasitism given that avoidance behaviours are likely widespread and could have considerable implications.

Our results show that parasites can have significant pre-encounter effects similar to that of predators by altering the foraging choices of larval amphibians. Forgoing foraging in areas where there is a threat of parasitism could have critical consequences as larval amphibians must achieve a minimum size in order to undergo metamorphosis, with small metamorphs also exhibiting increased vulnerability to predators and reduced fecundity [54,55,56]. Given that anthropogenic changes such as climate change and eutrophication have been linked to an increase in the production of the parasite infectious stage studied here (trematode cercariae), this may result in greater post- and pre-encounter impacts of parasitism on amphibians, which are already the most imperiled group of vertebrates worldwide [57,58,59]. As our findings support the importance of altered spatial use to avoid parasitism, habitat modifications that prevent foraging flexibility could also impact host anti-parasite defences. Pre-encounter effects arising from a fear, and avoidance, of pathogens may represent a general phenomenon occurring in other host-parasite systems and are in critical need of greater study given the ubiquity of parasites. Because parasitic species may actually outnumber free-living ones [60,61], many animals could encounter parasites more than predators. While the post-encounter effects of parasite infection can be considerable for individual hosts and populations [9,10,11], their pre-encounter effects are virtually unknown relative to predator-prey interactions. Integrating both predators and parasites into future studies will be important to better understand the ecology of fear and achieve a more comprehensive awareness of how natural enemies impact ecosystems.

## Supporting Information

S1 Table Tadpole foraging data. (XLSX)
